# Mitochondrial DNA alterations in precision oncology: Emerging roles in diagnostics and therapeutics

**DOI:** 10.1016/j.clinsp.2024.100570

**Published:** 2025-01-29

**Authors:** Alexis Germán Murillo Carrasco, Roger Chammas, Tatiane Katsue Furuya

**Affiliations:** a Centro de Investigação Translacional em Oncologia (LIM24), Instituto do Câncer do Estado de São Paulo (ICESP), Faculdade de Medicina da Universidade de São Paulo (FMUSP), São Paulo, Brazil; b Comprehensive Center for Precision Oncology, Universidade de São Paulo, São Paulo, Brazil

**Keywords:** Mitochondrial genome, Mutations, Biomarkers, Cancer, Precision oncology

## Abstract

Mitochondria are dynamic organelles essential for vital cellular functions, including ATP production, apoptosis regulation, and calcium homeostasis. Increasing research has highlighted the significance of mitochondrial DNA (mtDNA) content and alterations in the development and progression of various diseases, including cancer. The high mutation rate and vulnerability of mtDNA to damage make these alterations valuable biomarkers for cancer diagnosis, monitoring disease progression, detecting metastasis, and predicting treatment resistance across different tumor types. This review explores the emerging roles of mtDNA alterations in precision oncology, emphasizing their potential in theranostics. The authors explore the mechanisms by which mtDNA mutations contribute to tumorigenesis and therapy resistance, the impact of heteroplasmy in cancer biology, and the integration of mtDNA-based diagnostics with current therapeutic strategies. Additionally, the authors highlight the experimental tools and models currently used to investigate mtDNA alterations in cancer, including advanced sequencing technologies and animal models.

## Highlights

• Mitochondria regulate cellular functions, and mtDNA mutations can affect metabolism.
• mtDNA mutations are promising biomarkers for cancer detection and tumor progression.
• These mutations might influence therapy response, drug resistance, and patient outcomes.
• Tools and models advance precision oncology and mitochondrial-targeted treatments.

## Introduction

Mitochondria are dynamic organelles responsible for many functions, including regulation of apoptosis, redox balance, innate immunity, calcium regulation, metabolic intermediates biosynthesis, Oxidative Phosphorylation (OXPHOS), ATP synthesis, and many other vital signaling pathways essential for homeostasis maintenance inside the cells. Consequently, mitochondrial dysfunction has been linked to a wide range of diseases, including cancer.^1^, ^2^, ^3^

The energy metabolism of cancer cells is one of the most extensively studied areas correlating mitochondria and cancer. In 1927, Otto Warburg proposed that cancer cells preferentially use aerobic glycolysis for energy production, even in the presence of oxygen, rather than the conventional OXPHOS observed in normal cells.^4^ The “Warburg effect” highlighted a distinct metabolic characteristic of tumors, suggesting that mitochondrial dysfunction could play a crucial role in driving tumorigenesis.^4^

Mutations and alterations in mitochondrial DNA (mtDNA) have emerged as valuable biomarkers that can influence multiple aspects of cancer management, including diagnosis, monitoring disease progression, detecting metastasis, and predicting treatment resistance. This review aims to detail the role of the mtDNA changes in diagnosis and therapeutics, demonstrating how they can serve as powerful tools in personalized oncology.

## Mitochondrial genome

The first complete sequencing and structural organization of human mtDNA was reported in 1981.^5^ This circular, self-replicating, double-stranded genome consists of 16,569 base pairs and encodes 37 genes essential for OXPHOS, including 13 proteins, 22 transfer RNAs (tRNAs), and 2 ribosomal RNAs (rRNAs). Of the 13 proteins, mtDNA encodes 7 of the 45 subunits of complex I (ND1, ND2, ND3, ND4, ND4L, ND5, and ND6), one of the 11 subunits of Complex III (CYB), three of the 13 subunits of complex IV (CO1, CO2, and CO3), and two of the 17 subunits of complex V (ATP6 and ATP8).^3^

**Fig. 1:**
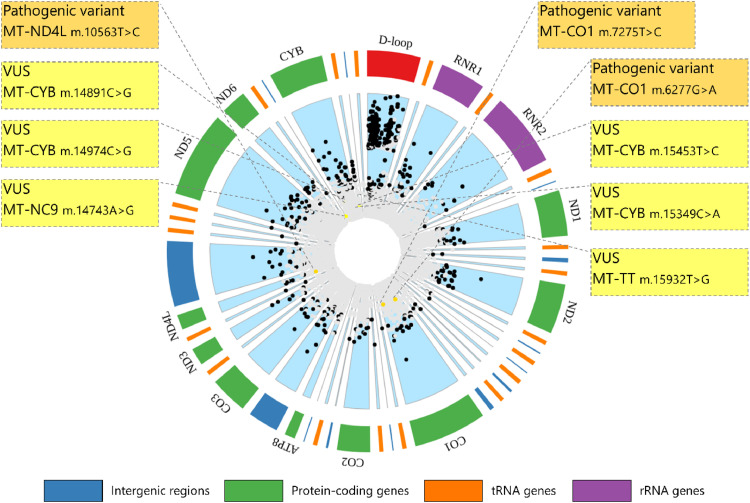
Representative map of mtDNA variations. The outer circle illustrates key mtDNA features, including protein-coding (green), tRNA (orange), and rRNA (purple) genes, along with intergenic regions (blue) avoiding overlapping regions. The inner circle presents a comprehensive map of all mtDNA variants documented in MITOMAP (up to October 2024), with a populational frequency above 0.0001 %. Variants with >15 references are highlighted in black. Additionally, cross-referencing with ClinVar (https://www.ncbi.nlm.nih.gov/clinvar/) identifies pathogenic variants and Variants of Uncertain Significance (VUS) in gold and yellow, respectively.

In addition to its coding regions, the mitochondrial genome contains a non-coding region of approximately 1 kb, known as the d-loop (Fig. 1), which plays a crucial role in the regulation of mtDNA replication and transcription.^5^ Besides the genes encoded by the mitochondrial genome, nuclear DNA (nDNA) encodes the remaining components of the OXPHOS system, as well as the machinery required for mtDNA replication, transcription, and other essential mitochondrial functions.^6^

In contrast to the nuclear genome, which is packaged into nucleosomal structures through its association with histones, mtDNA lacks histone support and is organized into distinct units called nucleoids.^7^ Each mitochondrion may contain numerous nucleoids, with each one housing several copies of mtDNA. These nucleoids are not randomly distributed but are strategically organized to support overall mitochondrial function and genomic integrity, such as mtDNA replication, repair, and segregation during cell division.^7^

mtDNA is highly susceptible to damage and its mutation rate is approximately 10 times higher than nDNA.^8^ This increased vulnerability can be attributed to several factors: mtDNA replicates more frequently and independently of the cell cycle, lacks protective histones, has limited DNA repair mechanisms, is continuously exposed to Reactive Oxygen Species (ROS) generated during OXPHOS, and possesses reduced antioxidant defenses. Additionally, the absence of introns in mtDNA increases the likelihood that mutations in coding sequences will have pathogenic effects.^9^, ^10^, ^11^, ^12^

As evidence of this mutation accumulation, MITOMAP, one of the most comprehensive databases for mtDNA variations, reports a total of 22,203 mtDNA variants (https://www.mitomap.org/MITOMAP, accessed in October 2024). Among them, 6510 variants exhibit a global frequency above 0.0001 % (Fig. 1). While these variants are distributed across the entire mtDNA genome, D-loop mutations are particularly frequent. Though this observation could be biased by the large number of studies focusing specifically on this region, it suggests a role in haplotype classification. On the other hand, coding region mutations are less characterized, potentially due to the challenges posed by heteroplasmy and limitations in the sensitivity of current detection systems.

Heteroplasmy refers to the coexistence of both normal and mutant mtDNA copies within a single cell.^13^ This phenomenon arises because, unlike nDNA mutations that are inherited uniformly by all progeny cells, mtDNA mutations are restricted to individual organelles.

Then, mtDNA mutations may either pre-exist at low heteroplasmy levels in normal tissues or arise *de novo*. Initially, low-heteroplasmy mutants are often phenotypically masked. However, a small subset of these cellular mtDNAs may drift to higher levels of heteroplasmy.^14^ Cellular dysfunction or disease phenotypes typically arise when the proportion of mutant mtDNA surpasses a critical threshold, which varies depending on the specific mutation and tissue type.^15^ Certain mtDNA mutations that provide selective advantages to cancer cells in specific environments can even shift from a state of heteroplasmy towards homoplasmy, where mutant mtDNA becomes dominant in tumor cells.^16^ As a result, these mutations may contribute to accelerated tumor growth, facilitate distant metastasis, and potentially confer resistance to treatment.^14^

Another significant challenge is that mtDNA mutations have been identified as part of the normal aging process,^17^ which can be easily confounded with tumor-related effects in elderly patients. This accumulation of age-related mutations, combined with the presence of heteroplasmy within cells, reveals significant challenges for experimental design and data interpretation when investigating the role of mtDNA as potential drivers or passengers in cancer risk and progression.

### mtDNA and cancer diagnosis

Numerous epidemiologic studies have shown significant correlations between germline mtDNA haplogroups and cancer risk and progression in certain population groups, as reviewed in other studies.^14^^,^^18^ These haplogroups represent specific combinations of germline mtDNA polymorphisms shared by different population groups. They are named sequentially in order of discovery using letters from A to Z, with deeper refinements denoted by additional numbers and letter combinations.^19^ A variety of mitochondrial haplogroups have been associated with varying risks for different types of cancers, such as the T-haplogroup for colorectal cancer (CRC),^20^ the M for breast cancer,^21^ the U for prostate and renal cancer,^22^ and the D4a for thyroid cancer.^21^ Other common mtDNA variants, whether linked to specific haplogroups or not, may also influence cancer risk.^14^^,^^18^ Some mtDNA alterations (i.e., T195C) have been associated with multiple different cancers, while others (i.e., T16189C) appear to be cancer-type specific.^18^ A better understanding of these variants and haplotype predispositions could facilitate more effective cancer screening and prevention strategies. However, establishing clear cause-effect relationships in population studies remains challenging due to various confounding factors, including the small sample sizes, limited number of variants or haplogroups studied, population stratification, and polymorphisms that may exert opposite effects in different ethnic populations.

Besides the germline variants, somatic mtDNA mutations can significantly affect mitochondrial function and contribute to tumorigenesis. Several studies have identified mtDNA mutations as potential drivers for cancer development,^3^^,^^23^^,^^24^ suggesting that these mutations could serve as valuable biomarkers for cancer diagnosis.

A study demonstrated that the respiratory OXPHOS complexes are fundamental determinants of the mutational burden and functional consequence of somatic mtDNA mutations across various tumor lineages.^25^ Certain cancer types exhibited a significantly higher burden of mtDNA mutations than others. The study revealed that the complex I subunits were enriched with loss-of-function truncating mutations, while complex V subunits were broadly depleted of all classes of non-synonymous mutations. Complex III displayed an elevated rate of missense variants, whereas synonymous variants showed no preference.^25^

A high incidence of mutations in mtDNA coding genes was observed in both esophageal cancer cell lines and cancer tissue, with *ND5* and *CYB* exhibiting the highest mutation frequencies compared to other identified genes.^26^ In lung cancer, mutation rates have been shown to be approximately 100 times higher than in normal tissues, with non-synonymous mutations occurring more frequently in the *ATP6* and *ND3* genes.^27^

It is well established that coding mtDNA mutations can disrupt protein function and impair the OXPHOS system. However, a high mutation rate has also been observed in non-coding regions, such as the D-loop region and tRNA genes,^16^ adding complexity to the interpretation of the functional impact of mtDNA mutations on cellular processes. For instance, five mt-tRNA mutations have been identified as likely pathogenic, potentially impairing mitochondrial function in breast cancer tissues.^28^

Yuan et al. (2020) conducted a comprehensive molecular analysis of mitochondrial genomes across various human cancers. By analyzing a large dataset of cancer samples, the researchers identified a wide range of somatic mtDNA alterations, including substitutions, insertions and deletions (indels), copy-number changes, and structural variations.^29^ They also explored the functional impact of these alterations, uncovering associations between specific mtDNA mutations and distinct cancer phenotypes.^29^

A reduction in mtDNA copy number has also been observed in many cancer types by analyzing data from primary human cancers from The Cancer Genome Atlas (TCGA) consortium.^30^ The results suggested that reduced mtDNA abundance may be linked to suppressed mitochondrial activity in these tumors.^30^ Another study that extracted mtDNA information from Whole Exome Sequencing (WES) data from 943 samples identified 215 indels, predominantly heteroplasmic, with a significant concentration in the D-loop region.^31^

Moreover, the transfer and integration of mtDNA segments into nDNA (NUMTs), a process known as numtogenesis, has been proposed as a potential mechanism for cancer development.^32^ A study analyzing 587 pairs of Whole Genome Sequencing (WGS) data from cancer patients and their corresponding normal tissues identified 25 cancer-specific mtDNA integration events into the nDNA across 12 patients that were absent in the matched control samples.^33^ Yuan et al. (2020) have also described frequent somatic transfer of mtDNA into the nuclear genome, often linked to processes that drive structural variations in nDNA.^29^ These nuclear transfers can promote genomic instability and activate oncogenic pathways by integrating into gene-rich regions of chromosomes, disrupting tumor suppressor genes, activating oncogenes, or inducing chimeric gene fusions.^32^

### mtDNA and tumor progression and metastasis

Ongoing research has demonstrated that mtDNA mutations not only correlate with cancer susceptibility but also influence tumor progression and metastatic behaviors across different tissues.^34^^,^^35^ These findings suggest that specific patterns of mtDNA mutations might affect the ability of tumor cells to colonize and thrive in certain organs, shaping metastatic outcomes.

By transferring mitochondria from highly metastatic mouse tumor cell lines into poorly metastatic ones, Ishikawa et al. (2008) observed a marked increase in tumor aggressiveness. The study identified specific mtDNA mutations (G13997A and 13885insC in the *ND6* gene) in complex I of the respiratory chain, which led to increased ROS production, linking mitochondrial dysfunction to metastatic potential.^36^ Similarly, the pathogenic mtDNA G10398A variant in the *ND3* gene has been shown to alter NADH dehydrogenase activity, increase the production of ROS, confer resistance to apoptosis, and enhance tumorigenicity both in vitro and in vivo.^37^ Conversely, a study demonstrated that complex I mtDNA mutations (C12084T and A13966G in the *ND4* and *ND5* genes, respectively) could drive high metastatic potential in breast cancer cell lines, independent of ROS-mediated pathways.^38^

In bladder cancer, specific mutations in the *CYB* gene, which is part of the complex III, contributed to enhanced tumor growth and progression, as demonstrated by in vitro and in vivo experiments.^39^ Another study showed that the introduction of the 13885insC mutation in *ND6*, enhanced distant metastasis in low-metastatic lung carcinoma cells. This process was linked to the overexpression of metastasis-related genes, metabolic reprogramming, increased tumor angiogenesis, and the acquisition of resistance to stress-induced cell death.^40^

Furthermore, somatic mtDNA mutations were identified in bone metastases of prostate cancer patients, where they were associated with enhanced survival and adaptation of cancer cells within the bone microenvironment. These findings emphasize the role of the bone niche in shaping the genetic landscape of metastatic prostate cancer and highlight the need to understand mtDNA alterations in the context of tumor microenvironments for the development of targeted therapeutic strategies.^41^

In addition, Schubert et al. (2020) demonstrated that mtDNA mutations can contribute to tumor heterogeneity by investigating Head and Neck Squamous Cell Carcinoma (HNSCC) samples and related Lymph Nodes (LNs).^42^ The samples, primarily representing advanced tumor stages, revealed a correlation between tumor progression and the accumulation of mtDNA mutations. Moreover, the detection of tumor-specific mtDNA mutations in affected LNs suggested a link between tumor cell dissemination and a high mtDNA mutation detection rate, emphasizing their potential role in cancer metastasis.^42^

Studies have demonstrated that mtDNA containing metastasis-promoting mutations from highly metastatic lung cancer cells can be transferred to low-metastatic cells and stromal cells, such as Cancer-Associated Fibroblasts (CAFs), macrophages, and cytotoxic T-cells, via Extracellular Vesicles (EVs). This transfer has been shown to contribute to tumor progression, enhance metastatic potential, and alter the phenotypes of stromal cells within the tumor microenvironment.^43^

A study in lung and colon cancer supported the concept of inherited susceptibility to metastasis. The study showed that specific nonsynonymous mtDNA variants in mitochondrial ND genes were associated with an increased likelihood of metastatic spread in these cancer types.^40^ An interesting aspect highlighted by this study was that homoplasmic states were less prevalent in metastatic lesions than in nonmetastatic cancer cells, suggesting that mutations in ND genes do not need to be homoplasmic to exert their pathogenic effect. Authors suggest that the more pathogenic a mutation is, the more likely it is to exist in a heteroplasmic state within cells, as long as the mutant loads surpass the pathogenic threshold.^40^ Inherited mitochondrial haplotypes have also been shown to influence mammary cancer latency and metastatic efficiency in an oncogenic driver-dependent manner.^44^ Furthermore, mtDNA mutations can also be prognostically meaningful in cancer. Particularly, pathogenic mtDNA mutations in CRC have been associated with better clinical outcomes and enhanced overall survival.^25^

### mtDNA and response to therapy

mtDNA alterations have been shown to significantly impact cancer therapy responses and outcomes.^45^ Therefore, understanding the mtDNA alterations and their influence on key mitochondrial functions such as OXPHOS, cellular energy metabolism, ROS production, and apoptosis regulation, could improve the effectiveness of cancer therapies, improve cancer patient outcomes, and provide opportunities for personalized treatment strategies.

The mitochondrial genome has been shown to play a critical role in determining the sensitivity of cancer cells to therapeutic agents. A study demonstrated that tumor cells lacking mtDNA exhibited a significant resistance to cell death induced by adriamycin and photodynamic therapy.^46^ Moreover, a recent study demonstrated that generating truncating mutations in the *Nd5* gene by mtDNA base editing induced a Warburg-like metabolic shift, reshaped tumor microenvironments, and enhanced antitumor immune responses in murine melanoma models. Tumors exhibiting high mtDNA mutation heteroplasmy showed better checkpoint blockade therapy responses compared to mtDNA wild-type cancers, highlighting the role of mtDNA mutations as functional regulators of cancer metabolism and potential therapeutic targets.^47^

Numerous reports have suggested that mtDNA variations can be induced by radiotherapy and chemotherapy.^45^ Increased point mutations and deletions have been reported in samples from patients treated with these modalities compared to control subjects.^48^ Additionally, a reduction in mtDNA content was reported in the salivary rinses of HNSCC cancer patients after postoperative radiation therapy.^49^

A case report described the emergence of an mtDNA mutation in the *ND4* gene after paclitaxel-carboplatin treatment in ovarian carcinoma, which was absent in the tumor tissue before the treatment, suggesting that chemotherapy may have induced or selected for this mutation, potentially contributing to resistance.^50^ Another study found that chemotherapy significantly increased the frequency of heteroplasmic mtDNA mutations in primary leukemia cells from chronic lymphocytic leukemia patients who had undergone prior treatment, compared to untreated patients, and these mutations were associated with increased ROS generation.^51^ Additionally, patients who were resistant to conventional therapies exhibited higher mutation rates compared to those who responded to treatment, suggesting a link between mtDNA mutations and drug resistance.^51^

On the other hand, a study found that mitochondrial mutations in children treated with chemotherapy for pediatric cancer did not increase mtDNA mutation load, as secondary leukemias showed no increase in mtDNA substitutions than primary leukemias from the same patients.^17^ Additionally, in vitro treatments with chemotherapy, antiviral drugs, or X-Ray did not increase mutation load or mtDNA copy number in clonally expanded cord blood cells from healthy donors, possibly due to mitochondrial genomes' inherent resistance, clearance of damaged mitochondria, or treatment-induced mutations existing at undetectable heteroplasmy levels.^17^

A study demonstrated that breast cancer cell lines with lower mtDNA copy numbers were more sensitive to anthracycline-based chemotherapy and produced higher levels of ROS, while patients with elevated mtDNA content experienced lower disease-free survival rates.^52^ Furthermore, decreased mtDNA content correlated with improved outcomes in breast cancer patients undergoing the same chemotherapy.^53^ This increased sensitivity could be due to higher vulnerability to mitochondrial damage, suggesting that mtDNA levels could potentially serve as biomarkers for predicting the efficacy of anthracycline-based therapies in breast cancer patients.^52^^,^^53^

Taken together, both chemotherapy and radiotherapy seem to be influenced by mtDNA alterations. Additionally, combined therapies integrating conventional treatments with drugs targeting mitochondrial functions could be explored as promising therapeutic strategies for cancer patients. Our research group has previously described the potential of metformin (Glucophage), a drug commonly used for type II diabetes that inhibits complex I and leads to a mitochondrial malfunction, as an adjuvant to cisplatin-based therapy in lung cancer cells in a p53-dependent manner.^54^

In addition, a study indicated that voluntary exercise could mitigate the effect of *PolgA* suppression, which is responsible for encoding mtDNA polymerase, decreasing the mtDNA mutation load, improving locomotor activity, and alleviating symptoms such as alopecia and kyphosis in a murine model,^55^ which opens new questions and hypotheses in cancer research where the effect of voluntary exercise is also a matter of study.^56^

### mtDNA detection by liquid biopsy

Significant efforts have been directed toward understanding cancer molecular profiling for precision oncology through non-invasive methods, such as liquid biopsy. This approach assesses a wide range of tumor-derived entities including Circulating Tumor Cells (CTCs) shed by both primary and metastatic tumors, cell-free circulating tumor DNA (ctDNA), tumor-derived EVs, Tumor-Educated Platelets (TEPs), and circulating cell-free RNA (cfRNA).^57^

Recent studies have highlighted the clinical value of cell-free mtDNA (cf-mtDNA) as a biomarker for cancer predisposition, screening, treatment guidance, prognosis assessment, and drug resistance detection.^58^ Therefore, cf-mtDNA could have the potential to make a substantial impact on the advancement of non-invasive cancer diagnostics and personalized treatment strategies.

A study showed that the fraction of tumor-derived mtDNA in plasma may serve as a biomarker correlated with tumor load.^59^ Also, its levels varied depending on cancer type, with increased abundance observed in patients with cholangiocarcinoma, CRC, liver, pancreatic, or prostate cancer compared to healthy individuals.^59^ Moreover, the detection of *ND1* content and specific variants in cf-mtDNA was demonstrated to be associated with response to chemotherapy and progression in CRC patients.^60^

It has been demonstrated that tumor-derived cf-mtDNA exhibited a higher detection rate and greater copy number compared to nuclear cfDNA plasma, cerebrospinal fluid, and urine from patient-derived orthotopic xenograft models of glioblastoma.^61^ This indicated that cf-mtDNA could be more sensitive than nuclear cfDNA for detecting and monitoring tumor burden.

For certain primary tumor sites, there is ongoing discussion about whether local liquid biopsy sources could be more suitable than systemic options such as plasma. Notably, a study demonstrated that urine samples are more effective for assessing the diagnostic potential of aberrant fragmentation and mutation profiles of cf-mtDNA in renal cell carcinoma (RCC) and CRC patients.^62^ Furthermore, another study evaluated a saliva-based liquid biopsy as a noninvasive source of cf-mtDNA for the early detection of HNSCC.^63^

Moreover, a study analyzing cfDNA from plasma samples of Hepatocellular Carcinoma (HCC) and prostate cancer patients revealed an inverse correlation between the length of mt-cfDNA fragments and both tumor size and circulating tumor DNA concentration.^64^ These findings suggest that monitoring the size of mt-cfDNA fragments in cancer patients could also be a valuable tool for estimating tumor burden and tracking cancer progression.^64^ Additionally, a study found that distinct profiles of genetic diversity distributed across the entire mtDNA from blood samples could accurately differentiate HCC patients from healthy controls, indicating their potential as biomarkers for HCC replacing analyses focused on specific mutations.^65^

Additionally, a study demonstrated the presence of larger mtDNA fragment sizes in EVs from patients with HCC, suggesting a complementary diagnostic value for the vesicular component of cf-mtDNA.^66^ Following research on EV-mtDNA in urogenital tumors, another study proposed that mtDNA within exosomes, a subset of EVs, could serve as a promising biomarker for assessing RCC aggressiveness.^67^

### Tools for studying the role of mtDNA mutations in cancer

To investigate the role of mtDNA variants in cancer, researchers have employed a range of advanced tools, including genomic sequencing techniques and functional assays. These approaches often integrate in vitro and in vivo models to assess the impact of specific mtDNA mutations on cancer hallmarks.

The emergence of high-throughput Next-Generation Sequencing (NGS) has significantly enhanced the accessibility of mtDNA sequence data for cancer research. Both WES and WGS have been widely used to explore mtDNA variations in cancer. However, careful interpretation is essential due to potential issues such as polymerase chain reaction (PCR) amplification errors, sequencing inaccuracies, and contamination with NUMTs.^14^

Moreover, tumors consist of heterogeneous compositions that include potentially distinct mtDNA genotypes from malignant, immune, and stromal cells. This diversity complicates the interpretation of heteroplasmy levels using conventional bulk sequencing methods, due to the presence of various malignant and non-malignant cell populations and the cell-to-cell variation in heteroplasmy levels within each population.^68^ To fully understand the selective pressures acting on these heterogeneous cell populations with diverse mtDNA genotypes, recent studies have focused on accurately quantifying heteroplasmy at the high-resolution single-cell level.^69^

Moreover, functional studies are required to complement the mtDNA genomic characterization. To investigate the functional significance of the mtDNA variants, researchers have developed mouse models that provide controlled environments to study the precise interactions between mitochondrial and nuclear genomes. These models help to elucidate the specific roles of mtDNA mutations and their influence on tumor behavior and help to better understand how mitochondrial genetics contribute to cancer initiation, growth, progression, and metastasis.^18^

The Cytoplasmic Hybrid (Cybrid) model involves the transfer of cytoplasm containing a foreign mitochondrial genome into nucleated cells that have the mtDNA depleted. The cybrid models have allowed researchers to investigate in vitro and in vivo effects of specific mtDNA mutations in cancer cell lines and tumors with an identical nuclear background.^36^^,^^45^

Mito-mice were among the first mouse models developed to study the role of mtDNA mutations in vivo. This was achieved by enucleating cybrid cells carrying an mtDNA mutation of interest, followed by fusing the resulting cytoplast with a pronuclear stage embryo using electroporation and implanting into a pseudo-pregnant female.^70^ Researchers have generated transmitochondrial mito-mice with a homoplasmic G13997A mutation in the *ND6* gene, which resulted in defects in respiratory complex I and lactate overproduction.^71^

The Mitochondrial-Nuclear Exchange (MNX) models were first described in 2013. This approach used nuclear transplantation technology to create mice with nuclear genomes from one inbred strain and mtDNA from a different inbred strain.^72^ Using the MNX model, researchers have investigated the impact of mitochondrial genetics on breast cancer tumorigenicity and metastatic potential. Their study revealed that variations in mtDNA regulated both the development of breast cancer tumors and their ability to spread.^34^ It has also been demonstrated that mitochondrial genomic backgrounds resulted in distinct nDNA methylation patterns and differential expression of nuclear-encoded genes, affecting cellular processes related to cancer.^73^

Moreover, researchers have developed a new generation of mice harboring mtDNA somatic mutations, known as mtDNA-mutator mice, which exhibit a targeted dysfunction in the *PolgA* gene. A study showed that aged individuals with this mutation can evade mitophagy-related processes, leading to a poor prognosis due to the continuous accumulation of mtDNA somatic mutations.^74^

Recent advancements in gene-editing technologies, such as CRISPR/Cas9, have been explored to enable the precise introduction or correction of mtDNA mutations, as reviewed elsewhere.^75^^,^^76^ However, editing mtDNA remains challenging due to the difficulty in targeting the necessary machinery within mitochondria, leading to incomplete editing and resulting in heteroplasmy, confounding the interpretation of the results. Additionally, mtDNA and nDNA share homologous sequences, complicating their differentiation during sequencing. Another challenge is the intricate mitochondrial-nuclear crosstalk, making it difficult to separate their effects as independent variables during analysis.^77^

## Conclusions and perspectives

**Fig. 2:**
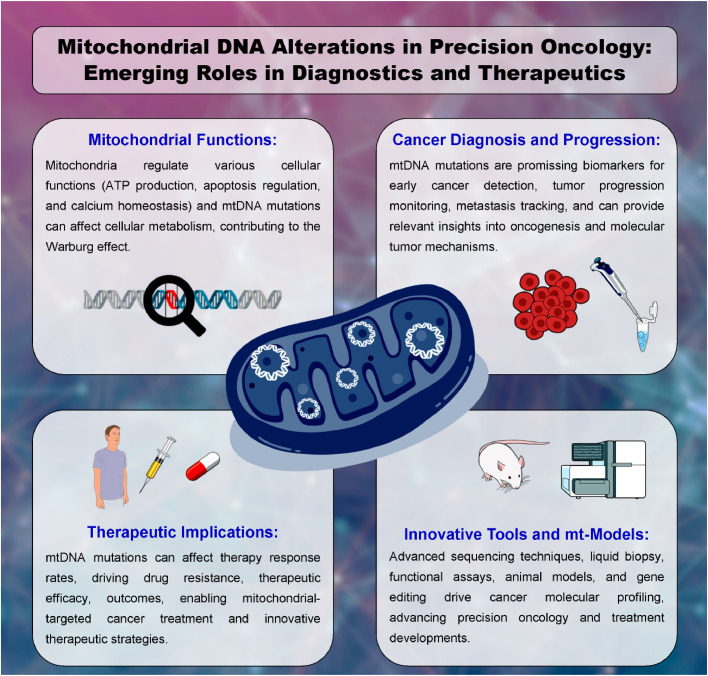
Graphical abstract providing a concise overview of the main topics covered in this review. Individual elements were downloaded from BioArt (https://bioart.niaid.nih.gov/) and Pixabay (https://pixabay.com/) sources.

The role of mtDNA alterations is increasingly recognized in cancer diagnosis and patient follow-up. This review compiles evidence indicating that mtDNA alterations can serve as key biomarkers for cancer risk, aggressiveness, metastasis, and therapy resistance due to their influence on critical cellular pathways, such as metabolism and apoptosis. While heteroplasmy complicates the detection of mtDNA mutations and challenges the sensitivity of affordable technologies, recent technological advances, such as high-resolution single cell mtDNA sequencing, are unraveling their complex contributions to cancer biology. Furthermore, the detection of circulating cf-mtDNA in liquid biopsies is emerging as a promising tool. These advanced methodologies offer new perspectives for functional studies involving preclinical mtDNA-based models to further investigate mitochondrial pathways, contributing to theranostic strategies that can potentially target and modulate cancer progression and treatment resistance. A visual summary of these approaches is shown in Fig. 2.

## Abbreviations

cf-mtDNA, cell-free mtDNA; CRC, colorectal cancer; cybrid, cytoplasmic hybrid; EVs, extracellular vesicles; HCC, hepatocellular carcinoma; HNSCC, head and neck squamous cell carcinoma; indels, insertions and deletions; LNs, lymph nodes; MNX, mitochondrial-nuclear exchange; mtDNA, mitochondrial DNA; nDNA, nuclear DNA; NGS, next-generation sequencing; OXPHOS, oxidative phosphorylation; PCR, polymerase chain reaction; RCC, renal cell carcinoma; ROS, reactive oxygen species; rRNA, ribosomal RNAs; TCGA, The Cancer Genome Atlas; tRNA, transfer RNAs; VUS, variants of uncertain significance; WES, whole exome sequencing; WGS, whole genome sequencing.

## Funding

This research did not receive any specific grant from funding agencies in the public, commercial, or not-for-profit sectors.

## CRediT authorship contribution statement

**Alexis Germán Murillo Carrasco:** Conceptualization, Formal analysis, Investigation, Visualization, Methodology, Software, Writing – original draft, Writing – review & editing. **Roger Chammas:** Conceptualization, Supervision, Visualization, Writing – review & editing. **Tatiane Katsue Furuya:** Conceptualization, Supervision, Formal analysis, Investigation, Visualization, Methodology, Writing – original draft, Writing – review & editing.

## Declaration of competing interest

The authors declare no conflicts of interest.
